# Targeting Protein Kinases to Protect Beta-Cell Function and Survival in Diabetes

**DOI:** 10.3390/ijms25126425

**Published:** 2024-06-11

**Authors:** Stéphane Dalle

**Affiliations:** Institut de Génomique Fonctionnelle, Université de Montpellier, Centre National de la Recherche Scientifique (CNRS), Institut National de la Santé et de la Recherche Médicale (INSERM), 34094 Montpellier, France; stephane.dalle@igf.cnrs.fr; Tel.: +33-4-3435-9203

**Keywords:** diabetes, pancreatic beta-cell, insulin secretion dysfunction, apoptosis, kinases, pharmacological inhibitors, therapeutic strategies

## Abstract

The prevalence of diabetes is increasing worldwide. Massive death of pancreatic beta-cells causes type 1 diabetes. Progressive loss of beta-cell function and mass characterizes type 2 diabetes. To date, none of the available antidiabetic drugs promotes the maintenance of a functional mass of endogenous beta-cells, revealing an unmet medical need. Dysfunction and apoptotic death of beta-cells occur, in particular, through the activation of intracellular protein kinases. In recent years, protein kinases have become highly studied targets of the pharmaceutical industry for drug development. A number of drugs that inhibit protein kinases have been approved for the treatment of cancers. The question of whether safe drugs that inhibit protein kinase activity can be developed and used to protect the function and survival of beta-cells in diabetes is still unresolved. This review presents arguments suggesting that several protein kinases in beta-cells may represent targets of interest for the development of drugs to treat diabetes.

## 1. Introduction

Massive death of pancreatic beta-cells by autoimmune reaction and reduction in beta-cell mass are major features of type 1 diabetes (T1D) [1,2,3,4]. In T1D, death of beta-cells is due to the infiltration of mononuclear cells, which leads to local and high concentrations of pro-inflammatory cytokines and chemokines in pancreatic islets [1,2,3]. Dysfunction of insulin secretion by beta-cells is a key event in the development of type 2 diabetes (T2D) [5,6,7,8]. A loss of beta-cell mass due to apoptotic death mechanisms has also been proposed as being critical for T2D [9,10]. Chronic exposure to hyperglycemia (glucotoxicity), certain high-concentration fatty acids (lipotoxicity), and deposits of islet amyloid polypeptide are well described to induce dysfunction and apoptotic death of beta-cells during T2D [11,12,13,14,15]. Chronic exposure to low-grade inflammation was also proposed to participate and cause beta-cell dysfunction and apoptotic death during T2D [16,17].

**Figure 1:**
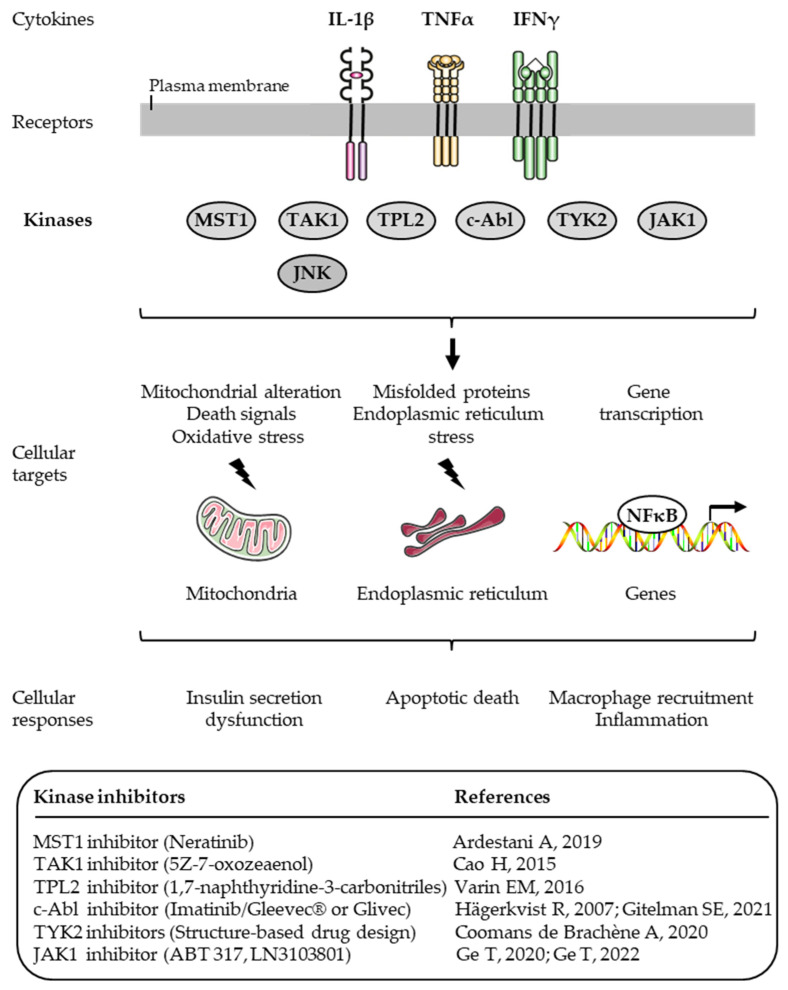
Targeting kinases to protect beta-cell function and survival from inflammation. Insulin secretion dysfunction, apoptotic death, inflammation and macrophage recruitment induced by pro-inflammatory cytokines occur through the activation of several intracellular kinases (MST1, TAK1, TPL2, c-Abl, TYK2, JAK1, and JNK) [29,30,31,32,33,34,35,36]. In beta-cells, activation of these kinases induces adverse effects including mitochondrial alterations, release of death signals from mitochondria, oxidative stress, ER stress, regulation of pro-inflammatory gene transcription through NFκB signaling. Targeting these kinases by pharmacological inhibition represents a strategy to prevent insulin secretion dysfunction and apoptotic death of beta-cells.

Destruction of beta-cells by immune reactions is a key characteristic of the pathogenesis of T1D [1,2,3,4]. Pro-inflammatory cytokines, such as interleukin-1β (IL-1β) and tumor necrosis factor-α (TNF-α), which are produced and released by immune cells that invade pancreatic islets, promote beta-cell dysfunction and apoptotic death [1,2,18]. Islet transplantation is now recognized as an effective treatment for some patients with T1D [19,20]. However, a significant loss of pancreatic islet mass occurs during and after transplantation [20,21], due to apoptotic death involving distinct mechanisms such as instant blood-mediated inflammatory response and the release of various cytokines, including IL-1β, TNF-α, and interferon-γ (IFN-γ) [20,21,22]. Chronic, systemic, low-grade inflammation was also proposed to be a participant in beta-cell dysfunction and death, and the pathogenesis of T2D [16,17]. Apoptosis and dysfunction of beta-cells induced by pro-inflammatory cytokines occur through the activation of intracellular protein kinases, mitochondrial alterations and release of death signals, endoplasmic reticulum (ER) stress, and regulation of gene transcription. Mitogen-Activated Protein Kinases (MAPKs) such as c-Jun N-terminal kinases (JNKs) are activated by pro-inflammatory cytokines and trigger the release of mitochondrial death and ER stress signals [1,2,23]. The nuclear factor-kappa B (NFκB) transcription factor is well described to mediate the deleterious effects of pro-inflammatory cytokines [24,25,26,27]. The active transcriptional subunit p65 of NFκB is sequestered and inactivated in the cytosol by the inhibitor of NFκB (IκB). Exposure to IL-1β and TNF-α induces a phosphorylation-dependent degradation of IκB through the activation of the IκB kinase (IKKβ), promoting the nuclear translocation of the active transcriptional subunit p65, and subsequent transcription of pro-inflammatory genes [26,27]. Inhibition of these signaling pathways by the use of inhibitors represents a strategy to prevent apoptotic death and dysfunction of beta-cells induced by pro-inflammatory cytokines for both types of diabetes, and in the context of islet transplantation [2,16,17,22,28] (Figure 1).

**Figure 2:**
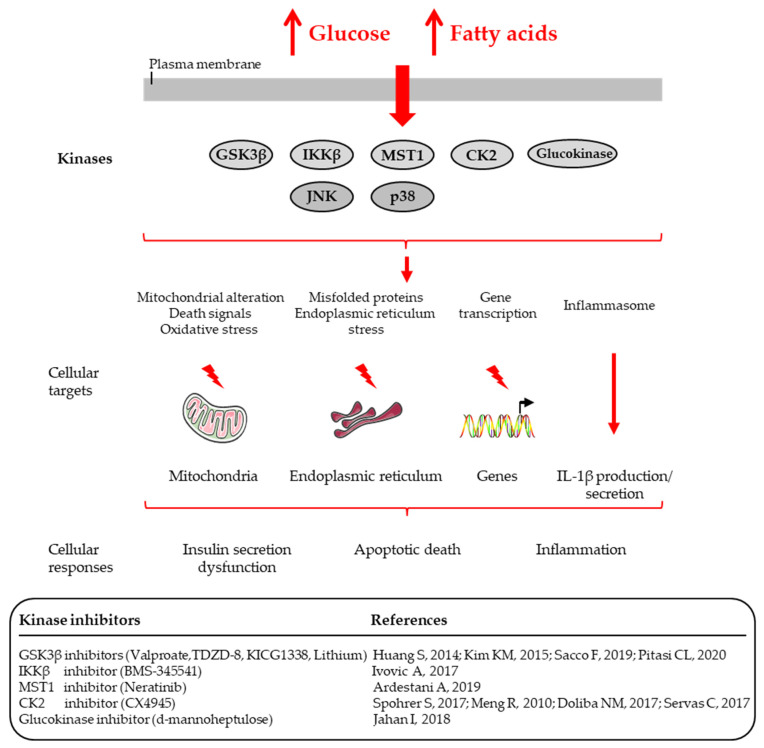
Targeting kinases to protect beta-cell function and survival from glucotoxicity and glucolipotoxicity. Insulin secretion dysfunction, apoptotic death, IL-1β production and secretion occur through the activation of intracellular kinases (GSK3β, IKKβ, MST1, CK2, JNK, p38) [29,42,43,44,45,46,47,48,49,50,51]. Activation of these kinases induces adverse effects including mitochondrial alterations, release of death signals from mitochondria, oxidative stress, ER stress, regulation of gene transcription, and stimulation of the inflammasome. Targeting these kinases by pharmacological inhibition represents a strategy to prevent insulin secretion dysfunction and apoptotic death of beta-cells. Pharmacological inhibition of the glucokinase activity decreases glucose metabolism, and may protect beta-cells from glucotoxicity.

Progressive loss of beta-cell function and mass characterizes T2D [5,6,7,8,9,10]. Chronic exposure to hyperglycemia (glucotoxicity), certain high-concentration fatty acids (lipotoxicity), and deposits of islet amyloid polypeptide are widely demonstrated to be involved the dysfunction and death of beta-cells [11,12,13,14,37,38]. Chronic exposure of beta-cells to hyperglycemia impairs insulin secretion and induces apoptotic death [11,12,13,14,37,38]. Synergistic adverse effects of high concentrations of glucose and saturated fatty acids on beta-cell function and survival have been reported (for reviews, see [14,39,40]). Chronic exposure of beta-cells to high concentrations of glucose and fatty acids such as palmitate (alone or in combination) impairs insulin secretion and induces apoptotic death (i.e., glucotoxicity, lipotoxicity, and glucolipotoxicity) by stimulation of intracellular stress kinases such as JNK and p38 MAPKinase, alteration of mitochondrial function, formation of reactive oxygen species (ROS), stress of ER, and intra-insular release of IL-1β pro-inflammatory cytokine [14,15,39,40,41]. Inhibitions of these pathways by pharmacological inhibitor approaches have been shown to be key strategies to prevent beta-cell dysfunction and apoptotic death (Figure 2).

In T1D and T2D, death and dysfunction of beta-cells contribute to the development of absolute or relative insulin deficiency. None of the antidiabetic drugs currently prescribed promotes the maintenance of a functional mass of endogenous beta-cells, revealing an unmet medical need. Protein kinases have become highly studied, and have been proposed as targets for drug development by the pharmaceutical industry. Drugs targeting and inhibiting protein kinases have been developed for the treatment of cancers. As examples, tyrosine kinase inhibitors are widely used in clinical oncology. Tyrosine kinase inhibitors target key mechanisms such as cell growth, invasion, metastasis, and angiogenesis (for reviews, see [52,53,54]). However, despite intensive research, the question of whether safe drugs that inhibit protein kinase activity can be designed and developed to protect beta-cell function and survival during T1D and T2D remains unresolved (Figure 1 and Figure 2; Table 1).

**Figure 3:**
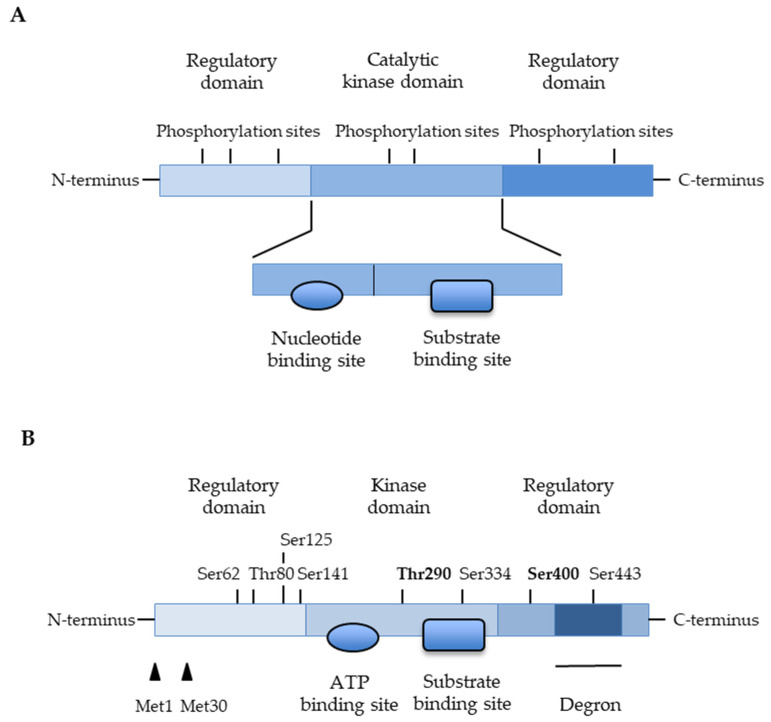
Structure of kinases. (**A**) Structure of kinases presents different domains: A catalytic domain usually composed of a larger α-helical domain; C-terminal regulatory domain, and N-terminal regulatory domain usually composed of a β-sheet domain. The N-and C-terminal domains can be linked by scaffold peptide forming a deep groove enabling nucleotide- and substrate-binding. The nucleotide-binding region can rotate into “on” and “off” conformations depending on the nucleotide-binding and kinase activation. Theses domains can display phosphorylation sites for activation, inactivation, degradation, or conformational changes. (**B**) Example of TPL2 kinase structure: TPL2 is expressed as 2 isoforms generated by alternative translation start sites (i.e., methionine1 (Met1), methionine30 (Met30)). The kinase domain is located in the center of the protein and flanked by N- and C-terminus regulatory domains. The C-terminus domain displays a degron sequence that targets TPL2 to the proteasome for degradation. Some phosphorylation sites are identified (Serine (Ser), Threonine (Thr)): Threonine 290 (Thr290) and serine 400 (ser400) are known to regulate kinase activity. The architecture of the TPL2 kinase domain is presented and illustrated in the review by Xu and collaborators [59].

Protein kinases are ubiquitous intracellular and cellular surface proteins with enzymatic properties. Protein kinases selectively catalyze protein phosphorylation by the transfer of a phosphate group from ATP, GTP, and other phosphate donors to the -OH group of amino acids (threonine, serine, and tyrosine) in protein substrates, allowing conformational changes from an inactive to an active form (for reviews, see [52,53,54]). The signal transduction is due to the reversible phosphorylation of proteins by modifying the structure and function. Protein kinases constitute a very extensive family of structurally bound enzymes that are involved in the control of cell growth, proliferation, differentiation, apoptotic death, cytoskeletal rearrangement, metabolic pathways, membrane transport, and motility [52,53,54]. Kinases usually have a catalytic domain with nucleotide and substrate binding sites, flanked by regulatory domains, inhibitor binding sites, and phosphorylation sites, and can undergo conformational rearrangements (Figure 3). To date, nearly 535 human protein kinases have been identified [53,54]. The two main classifications based on the enzymatic activities are the serine/threonine kinase proteins that phosphorylate proteins on serine/threonine residues, and the tyrosine kinase proteins that phosphorylate proteins on tyrosine residues [52,53,54]. Beyond their phosphorylation functions, protein kinases exhibit non-catalytic functions such as the formation of complex protein scaffolding and binding to DNA [58].

Genetic alteration and modification of the protein kinase functions are linked to cancer and immunological, neurological, cardiovascular, and metabolic diseases [52,53,54]. Therefore, protein kinases are considered to be important clinical targets for the treatment of various diseases. This review presents studies and arguments suggesting that several protein kinases in beta-cells may represent potential targets for the development of drugs to treat T1D and T2D (Figure 1 and Figure 2; Table 1).

## 2. Targeting Protein Kinases to Protect Beta-Cell Function and Survival from Inflammation

### 2.1. Targeting the Serine/Threonine Kinase Mammalian Sterile 20-like Kinase 1 (MST1)

Mammalian Sterile-20-like kinase (MST1), also known as serine/threonine-protein kinase 4 (STK4) or stress-responsive protein kinase-2 (KRS2), is an ubiquitously expressed serine/threonine kinase [60,61]. MST1 is described to be involved in cellular morphogenesis, proliferation, stress responses, chromatin condensation, and apoptotic death. MST1 induces cell death via the activation of various downstream targets such as the Hippo signaling pathway (large tumor suppressor 1 and 2 (LATS1/2)), forkhead box O (FOXO) family members, JNK, and caspase-3 activation [60,61]. Expression of MST1 was shown to be increased in beta-cells chronically exposed to pro-inflammatory cytokines and high glucose concentrations [62]. MST1 was further shown to be a key actor in cytokine-induced apoptotic death and beta-cell dysfunction [62,63]. Cytokines activated MST1, leading to the stimulation of JNK, thus promoting apoptotic pathways and dysfunction of insulin secretion. Activation of MST1 induced the degradation by the proteasome of the transcription factor duodenal homeobox 1 (PDX1), which controls insulin synthesis and beta-cell differentiation [62]. Invalidation of MST1 promoted the maintenance of normoglycemia by functional beta-cells and prevented diabetes [62]. The approved drug targeting the human epidermal growth factor receptor oncoproteins (EGFR and HER2), neratinib, was identified as a potent inhibitor of MST1. In vivo administration of neratinib protected beta-cells from the deleterious effects of the autoimmune process during T1D in NOD mice [29]. These studies support the design of MST1 inhibitors based on the structure of neratinib, with increased potency and selectivity for MST1 and safety profiles.

### 2.2. Targeting the Serine/Threonine Kinase Transforming Growth Factor-β Activated Kinase-1 (TAK1, or MAP3kinase 7)

The serine/threonine kinase transforming growth factor-β activated kinase-1 (TAK1, or MAP3kinase 7) is well described to stimulate JNK and NFκB signaling pathways, and to play an important role in innate and adaptive immune responses [64,65,66]. TAK1 is activated by multiple extracellular stimuli such as IL-1β, TNF-α, toll-like receptor (TLR) ligands, transforming growth factor-β (TGF-β), B cell receptor (BCR), and T cell receptor (TCR) ligands. Following activation, TAK1 phosphorylates and stimulates IKKβ and JNK, inducing the transcriptional activities of NF-κB and protein-1 (AP-1), and subsequent expression of genes involved in inflammatory processes and recruitment of immune cells [64,65,66]. Administration in vivo of 5Z-7-oxozeaenol (OZ), a potent and selective TAK1 and ATPase inhibitor, prevented systemic and pancreatic islet inflammation by decreasing production of pro-inflammatory cytokines and infiltration of lymphocytes [30]. Administration of OZ in vivo reduced diabetes in NOD mice and insulitis, preserved insulin secretory function, and inhibited JNK and NF-κB activation in beta-cells [30]. These studies suggest that strategies specifically targeting TAK1 without suppressing peripheral immune responses could be useful for treating T1D [30].

### 2.3. Targeting the Tumor Progression Locus 2 Kinase (TPL2, or MAP3kinase 8)

We reported that targeting MAP3kinase 8 or Tumor progression locus 2 (TPL2) prevents dysfunction and apoptosis of beta-cells [31]. TPL2 is a serine/threonine kinase (Figure 3) expressed in immune cells, and functions downstream of IKKβ. TPL2 is known to be activated by multiple stimuli including IL-1β, TNF-α, chemokines, and TLR ligands (for reviews, see [59,67]). We found that TPL2 is expressed in beta-cells and human pancreatic islets [31]. Expression of TPL2 was further found to be increased in beta-cells following chronic exposure to pro-inflammatory cytokines (i.e., IL-1β alone or IL-1β + TNF-α + IFNγ) [31]. Pharmacological inhibition of TPL2, using a naphtyridine compound acting as a specific reversible ATP-competitive inhibitor of TPL2 and developed for the treatment of rheumatoid arthritis and inflammatory bowel disease [68,69], prevented apoptotic death and dysfunction of beta-cells and human islets chronically exposed to IL-1β or several proinflammatory cytokines (IL-1β + TNF-α + IFNγ) [31]. Therefore, pharmacological inhibition of Tpl2 may be more efficient than inhibition of IL-1β or TNF-α alone. Multiple cytokines are likely present simultaneously within the islets of patients with T1D, exerting synergistic deleterious effects [1,2,18]. Instant blood-mediated inflammatory response occurring during the post-transplantation period destroys the transplanted pancreatic islets [22]. To protect beta-cells in T1D and during islet transplantation, the TPL2 kinase identified as a key mediator of deleterious effects of various cytokines would emerge as an interesting therapeutic target [31].

### 2.4. Targeting the Cellular Abelson Tyrosine Kinase (c-Abl)

The cellular Abelson tyrosine kinase (c-Abl) controls various downstream signaling pathways involved in cell proliferation, cell motility, and apoptotic death [70,71]. Imatinib mesylate (also known under the trade name as Gleevec^®^ or Glivec, Novartis, Basel, Switzerland), is a tyrosine kinase inhibitor which is well described to inhibit the c-Abl kinase, the platelet derived growth factor receptor (PDGFR), and the transmembrane receptor tyrosine kinase (c-kit) [70,71]. Imatinib is an oral chemotherapy drug successfully used in the clinic to treat chronic myeloid leukemia caused by Bcr-Abl oncogene and gastrointestinal stromal tumors caused by c-kit mutations [72,73]. Although imatinib inhibits a number of tyrosine kinases, it appears be selective to c-Abl, which is expressed and active in various types of cancers. Imatinib inhibits c-Abl by binding to the ATP pocket, which is located in the active site. This binding prevents the c-Abl activity, thus blocking phosphorylation of downstream cellular target proteins. Treatment of streptozotocin-injected mice with imatinib was reported to reduce the development of diabetes [32]. The use of imatinib decreased apoptotic death of human islets exposed to pro-inflammatory cytokines. Inhibition of the NFκB signaling pathway was proposed as the cellular mechanism of the anti-apoptotic action of imatinib [32]. The safety and efficacy of imatinib to preserve insulin secretion in patients with T1D was investigated and evaluated in a phase II clinical trial (NCT01781975) [33]. Importantly, imatinib treatment was shown to preserve beta-cell function in adults with recent T1D [33].

### 2.5. Targeting the Tyrosine Kinase 2 (TYK2)

Signaling pathways activated by interferon-α (IFN-α) were shown to be involved in the pathophysiology of T1D [74]. IFN-α and IFN-γ were recently reported to be key cytokines at the level of the pancreatic islet in human T1D, and contribute to the triggering and amplification of autoimmunity [74]. Type I IFN was shown to be expressed in human islets isolated from donors with T1D [75], and children genetically at-risk for the disease present a type I IFN transcriptomic signature in the circulation prior to the development of auto-antibodies [76]. Exposure of human beta-cells to IFN-α induced the release of chemokines, ER stress, human leukocyte antigen (HLA) class I overexpression, and apoptosis when combined with IL-1β [77]. These deleterious effects were mediated through the activation of two kinases, mammalian janus kinase 1 (JAK1) and tyrosine kinase 2 (TYK2), and the transcription factors, signal transducer, and activator of transcription 1 and 2 (STAT1 and STAT2). The TYK2/STAT2/IFN regulatory factor 9 (IRF9) signaling pathway was found to mediate the action of IFN-α in human beta-cells [77]. Using structure-based drug design approaches, pharmacological inhibitors of TYK2 were developed [34]. Designed inhibitors were found to act as allosteric inhibitors of the TYK2 kinase activity by binding to the JH2 pseudokinase domain [34]. The JH2-directed allosteric inhibition provided a high specificity for TYK2. These TYK2 inhibitors protected human beta-cells against the deleterious effects of IFN [34]. These studies provide a rationale for conducting future clinical trials for the treatment of T1D using TYK2 inhibitors.

### 2.6. Targeting the Mammalian Janus Kinase 1 (JAK1)

ABT 317, a mammalian janus kinase 1 (JAK1)-selective inhibitor [35], reduced IL-21, IL-2, IL-15, and IL-7 signaling in T cells, and IFN-γ signaling in beta-cells. Development of diabetes was reduced in NOD mice treated with ABT 317 [35]. ABT 317 blocks common γ chain cytokines in lymphocytes, and interferons in lymphocytes and beta-cells, and is thus more effective against diabetes pathogenesis than IFN-γ receptor deficiency alone. Authors proposed to use this class of inhibitory drug for the treatment of T1D [35]. Immune checkpoint inhibitors (i.e., antibodies that block the programmed death-1 receptor (PD-1)/programmed death receptor ligand 1 (PD-L1) interaction) are used in cancer treatments [55]. However, immune checkpoint inhibitor treatments are known to cause immune-related adverse events. T1D is described as a side effect of immune checkpoint inhibitor therapy for cancer [56]. Administration of LN3103801, a JAK1/JAK2 inhibitor, prevented anti-PD-L1-induced diabetes in NOD mice by blocking common gamma chain cytokine activities in T cells, and IFN-γ function on beta-cells [36]. These studies provide preclinical validations for the use of JAK inhibitors in checkpoint inhibitor-induced diabetes [36].

## 3. Targeting Protein Kinases to Protect Beta-Cell Function and Survival from Glucotoxicity and Glucolipotoxicity

### 3.1. Targeting the Glycogen Synthase Kinase-3β (GSK-3β)

The serine/threonine protein kinase glycogen synthase kinase-3 (GSK3) is ubiquitously expressed. GSK3 is involved in multiple pathways regulating glycogen metabolism, cell development, gene transcription, protein translation, cytoskeletal organization, cell cycle regulation, proliferation, and apoptotic death. The role of GSK-3β was investigated in beta-cells, and evaluated for the treatment of diabetes. Research found that the mood stabilizer valproate (valproic acid) is an inhibitor of GSK-3β kinase activity. Valproate treatment protected INS-1 beta-cells from apoptosis induced by palmitate [42]. TDZD-8, an inhibitor of GSK-3β, exerted an anti-apoptotic effect [42]. Administration of KICG1338, another inhibitor of GSK-3β, was reported to preserve beta-cell function in vivo [43]. Using high-sensitivity mass spectrometry-based proteomics analysis, GSK-3β was further found to be a key kinase involved in the signaling network controlling the beta-cell-specific transcription factor PDX1 [44]. In this study, GSK-3β was shown to be a kinase-inducing insulin secretion dysfunction following chronic exposure to hyperglycemia. Pharmacological inhibition of GSK-3β preserved the insulin secretion in response to glucose [44]. Goto–Kakizaki rats, a spontaneous nonobese model that develops T2D, were treated with infra-therapeutic doses of lithium, a widely used inhibitor of GSK3. The lithium treatment reduced the inflammation in the islets, partially restored the glucose-induced insulin secretion and reduced diabetes [45].

Glucocorticoids are commonly used for their anti-inflammatory and immunosuppressive properties [78]. However, it is well described that the use of glucocorticoids is associated with side effects such as diabetogenic effects [79]. Notably, activation of GSK-3β was reported to regulate the expression of the nuclear glucocorticoid receptor in beta-cells [57]. Using both genetic and pharmacological approaches, GSK-3β activation was found to mediate insulin secretion dysfunction and apoptosis induced by glucocorticoids in beta-cells [57]. The treatment of islets with GSK3 inhibitors, either lithium chloride or SB216763, significantly decreased the glucocorticoid-induced apoptotic death in pancreatic islet cells [57].

### 3.2. Targeting the Inhibitor of Nuclear Factor Kappa-B Kinase Subunit Beta (IKKβ)

Excessive production and accumulation of ROS induce oxidative stress. Oxidative stress is well known to play a key role in beta-cell dysfunction induced by fat (for reviews, see [14,39,40]). Oxidative stress activates IKKβ. Prolonged exposure of beta-cells to fatty acids, which induces oxidative stress and leads to insulin secretion dysfunction via the activation of IKKβ [46]. To investigate the role of IKKβ in the molecular mechanisms of insulin secretion dysfunction, a selective inhibitor of IKKβ (BMS-345541), which binds at an allosteric site of the enzyme and blocks NF-κB-dependent transcription, was used [46,80]. Treatment using BMS-345541 and deletion of IKKβ prevented the dysfunction of beta-cells in vitro and in vivo [46]. Ivovic A and colleagues demonstrated that IKKβ mediated insulin secretion dysfunction induced by fatty acids, and proposed the IKKβ/NFκB signaling pathway as a therapeutic target to prevent fatty acid-induced beta-cell dysfunction in vivo [46].

### 3.3. Targeting the Serine/Threonine Kinase Mammalian Sterile 20-like Kinase 1 (MST1)

The serine/threonine kinase MST1 was reported to be a key regulator of beta-cell apoptotic death and dysfunction induced by cytokines and chronic hyperglycemia [62,63]. Administration of neratinib, a potent inhibitor of MST1, was reported to improve beta-cell survival and to preserve beta-cell function in a *db/db* mouse model of T2D [29].

### 3.4. Targeting the Adenosine Monophosphate-Activated Protein Kinase (AMPK)

The serine/threonine adenosine monophosphate-activated protein kinase (AMPK) is composed of a catalytic α-subunit (α1 and α2 isoforms), a regulatory β-subunit (β1, β2 isoforms), and γ- (γ1, γ2, γ3 isoforms) subunits. AMPK is activated by an increased intracellular AMP/ATP ratio, and through threonine phosphorylation (Threonine 172 residue) on the activation loop of the α-subunit in response to hypoxia, ischemia, glucose deprivation, caloric restriction, or increased physical activity [81,82,83]. The binding to the γ-subunit of AMP and ADP is competitively inhibited by ATP. AMPK is thus described as a sensor of AMP/ATP or ADP/ATP ratios in cells, and as an important intracellular energy sensor [81,82,83,84,85,86,87]. Following activation, AMPK phosphorylates several proteins and enzymes, shifting metabolic pathways toward increased production of ATP, and inhibiting cellular processes consuming ATP [82,83,84,85,86,87]. AMPK was found to be expressed in beta-cells and human islets, and proposed to play a role in insulin secretion [88,89,90,91,92,93,94]. Using the pharmacological activator of AMPK, AICAR (AICA riboside, 5-aminoimidazole-4-carboxamide ribonucleotide), AMPK was investigated in beta-cells. Treatment of beta-cells with AICAR was shown to prevent AMPK inhibition induced by glucose and to increase insulin secretion. Moreover, pharmacological activation of AMPK was found to protect rat islet cells against glucolipotoxicity [93]. However, it should be noted that some reports using beta-cell lines or isolated islets demonstrated that the pharmacological activation of AMPK inhibited glucose-induced insulin secretion, while others indicated stimulatory effects or a lack of changes in insulin secretion. More studies are needed to identify the role of AMPK in beta-cells under physiological and pathological situations, and to potentially use AMPK modulators [88,89,90,91,92,93,94].

### 3.5. Targeting the Protein Kinase CK2 (Previously Called Casein Kinase 2 or CK-II)

Protein kinase CK2 is an ubiquitously expressed, constitutively active serine/threonine- and tyrosine kinase [95,96,97]. CK2 is formed of two catalytic α- or α’- and two non-catalytic β-subunits [95,96,97,98,99,100]. The β subunit of CK2 is an on–off regulator of the catalytic activity of CK2α, and regulates the thermostability, the substrate specificity, and the ability to attach and to penetrate cell membranes [101,102,103,104]. Studies reported an important role of CK2 in insulin secretion and beta-cell survival. Inhibition of CK2 by the selective CK2 inhibitor CX-4945 was shown to increase insulin secretion in response to glucose [47,105]. The transcription factor PDX-1 was found to be phosphorylated by CK2. This CK2 phosphorylation of PDX-1 resulted in a decreased insulin gene transcription [47,48,105]. Hence, active CK2 is proposed to act as a molecular repressor of insulin gene transcription. Notably, MST1 was found to be a CK2 substrate [49]. Inactivation of CK2 using CX-4945 was shown to protect beta-cells from apoptotic death and dysfunction induced by glucolipotoxicity [50]. These reports proposed that targeting CK2 using inhibitors might represent a novel therapeutic strategy for diabetes treatment. However, it is important to note that the CK2 inhibitors display high cell membrane penetrative properties without any cell specificity. The use of these CK2 inhibitors may favor the risk of major side effects.

### 3.6. Targeting the Glucokinase

Glucokinase (also known as hexokinase type IV) is the first enzyme in the glycolysis process, and is mainly expressed in the pancreas, liver, hypothalamus, and gastro-intestinal tract [106,107]. Expression of glucokinase is also found in the brain and the pancreatic alpha-cells, which synthesize and secrete glucagon [106,107]. Glucokinase is a particular kinase that phosphorylates glucose. Glucokinase exhibits a low affinity for glucose, and phosphorylates glucose to glucose-6-phosphate [106,107,108,109].

Glucokinase was found to be highly expressed in beta-cells, and to exert an essential role in the glucose metabolism and in insulin secretion [106,107,108,109]. Importantly, inactivating and activating mutations of glucokinase are well described to cause diabetes and hyperinsulinism, respectively [110,111,112]. Notably, activators of glucokinase were designed and developed as potential antidiabetic agents (for reviews, see [113,114,115,116]). Unfortunately, the use of glucokinase activators has failed as a therapeutic approach to preserve insulin secretion in diabetes [113,114,115,116,117,118]. Some glucokinase activators have been discontinued because of efficacy and safety reasons. As example, loss of the drug’s efficacy over time was observed (for reviews, see [113,114,115,116,117]). Paradoxically, reports suggested that decreasing glucose metabolism by inhibiting the activity of glucokinase within the beta-cells could be a successful approach to protect beta-cells against chronic hyperglycemia, and to preserve a functional beta-cell mass in diabetes [51,114,119,120,121]. Inhibition of glucokinase, by reducing glycolytic flux, was indeed reported to preserve beta-cell mass, reduce ER stress, preserve mitochondrial morphology and function, maintain a beta-cell phenotype, and insulin secretion in preclinical mouse models of monogenic diabetes and T2D [51,121,122]. Challenges facing the use of glucokinase inhibitors are finding inhibitors that specifically target glucokinase within the beta-cells, evaluating the degree to which glucokinase inhibition is relevant, and the positioning and timing of glucokinase inhibition treatment.

### 3.7. Targeting the Protein Kinase RNA-like Endoplasmic Reticulum Kinase (PERK)

Due to its importance in the dysfunction and apoptotic death of beta-cells, the ER stress is proposed as a promising therapeutic target for diabetes [39,40,41,123]. Chemical or pharmaceutical chaperones, such as phenylbutyrate and taurine-conjugated ursodeoxycholic acid derivative, stabilize protein conformation and are proposed to improve the folding capacity of ER (for review see [123,124]). Another proposed approach is to target the protein kinase RNA-like endoplasmic reticulum kinase (PERK). When activated by phosphorylation, PERK phosphorylates the eukaryotic initiation factor 2 alpha (eIF2α). The phosphorylation of eIF2α causes inhibition of protein translation, which relieves the workload of ER during cellular stress (for review see [123,124]). Studies using cell cultures, animal models, and human islets have identified an important role of PERK in the insulin secretion of beta-cells during embryonic development and adulthood. PERK was proposed to play a key role in beta-cell physiopathology and diabetes (for review see [123,124]). Some therapeutic strategies to reduce ER stress of beta-cells have been logically suggested using modulators of PERK activity [123,124,125,126,127].

## 4. Targeting Protein Kinase to Induce Beta-Cell Proliferation and Regeneration

The dual-specificity tyrosine phosphorylation-regulated kinase A (DYRK1A) is a kinase that phosphorylates proteins on serine/threonine. Activation of DYRK is obtained by phosphorylation of a tyrosine residue in the activation loop of the kinase (for review see [128]). Following activation, DYRK1A was reported to regulate various signaling pathways by activating or inactivating transcription and translation factors (RNA polymerase II CTD [129], Sprouty2 [130], DREAM complex [131], cAMP response element-binding protein (CREB) [132], and other proteins such as caspase-9 [133,134], Notch [135], and glycogen synthase [136]. DYRK1A was reported to be expressed in beta-cells [137,138,139]. Upregulation of DYRK1A in beta-cells promoted a beta-cell mass expansion through increased cell proliferation and size [137]. Studies revealed a role for DYRK1A kinase in human β-cell replication, proliferation, and function [138,139], and suggested the development of potential therapeutic strategies targeting DYRK1A using DYRK1A inhibitors [128,140,141,142,143]. Harmine and its derivatives are one of the most studied DYRK1A inhibitors [128,140,141,142,143].

## 5. Conclusions and Perspectives

To prevent the development of T1D and T2D, the preservation of a functional mass of beta-cells is essential. Our increased knowledge of the signaling pathways and networks involved in inflammation and nutrient stresses that induce dysfunction and death of beta-cells reveals the relevance of developing novel therapies targeting kinases to prevent diabetes.

This review identifies more than 12 different kinases that contribute to the dysfunction and death of beta-cells, and that are interesting targets for the development of drugs to treat diabetes. Among these kinases, there are tyrosine kinases and serine/threonine kinases, each found in distinct signaling networks, and each phosphorylating more likely different downstream substrates. These kinases are found within the signaling networks, which are well described to be deleterious following chronic exposure of beta-cells to pro-inflammatory cytokines or hyperglycemia. It is logical to hypothesize that the most interesting targets would seem to be those activated in the core of signaling networks engaged by both inflammation and hyperglycemia. Moreover, these targeted kinases should not participate in complex feedback loops where inhibition would lead to the activation of other deleterious signaling networks or block anti-inflammatory signaling pathways.

A complete characterization of the role of these kinases in their respective cellular networks, as well as their possible interrelationships, in beta-cells is now necessary to consider new options for the treatment of diabetes. It becomes necessary to accurately map these signaling networks in the diseased beta-cells. It can be hypothesized that mutations or expression levels of kinases in the diseased beta-cells may change the nature of these complex signaling networks that contribute to the disease phenotype. A comprehensive map of these signaling networks in the normal beta-cells is also necessary to understand possible changes and plasticity of these networks that operate in the diseased beta-cells. Obtaining these maps would allow the definition of a hierarchy between these kinases as well as their possible interrelationships. To date, a comprehensive map of the interconnected kinases, signaling networks, and plasticity, in healthy versus diseased-human beta-cells, is still missing.

The relevance of the use of kinase inhibitors to treat diabetes has been validated in vitro and in vivo in preclinical experimental models. Many kinase inhibitors were developed and tested in experimental models following the driven “one target-one drug” strategy. The efficacy of some inhibitors is based on their modes of action at the level of the target of interest, but can also be based on their modes of action at the level of other targets. Understanding the effects of an inhibitor’s action at the level of a targeted kinase within a signaling network is also essential in order to evaluate the efficacy and potential side effects. In parallel, another objective of this type of study is to determine the impact of the inhibitor used on all signaling networks.

Signaling networks tend to be redundant and are often relatively unaffected by the inhibition of a single kinase. In beta-cells, we may need to inhibit the activity of more than one type of kinase to achieve the desired therapeutic effects. Targeting multiple signaling pathways in beta-cells through the use of pharmacological agents with complementary mechanisms of action seems appropriate. The new antidiabetic medicine targeting both glucagon-like peptide-1 receptor (GLP-1R) and glucose-insulinotropic polypeptide receptor (GIPR) confirms this idea [144]. Kinase inhibitors used to reinforce beta-cell protection against the deleterious effects of inflammation and nutrient stresses may be used in combination with antidiabetic medicine targeting both GLP-1R and GIPR.

None of the available antidiabetic drugs promotes the maintenance of a functional mass of endogenous beta-cells, revealing an unmet medical need. Thanks to highly sophisticated analyses, such as mass spectrometry analyses combined with bioinformatics, kinases and signaling networks involved in the dysfunction and/or death of beta-cells may be better characterized. In beta-cells, the discovery process is still at a preclinical level for all kinase inhibitors discussed in the review, with the exception of imatinib. Future large-scale and chemical analyses and human clinical trials are also needed to assess the suitability of some of these inhibitors for their specificity and selectivity, pharmacokinetic properties, efficacy, safety, dose, and/or durability of treatment. Together, these different analyses will make it possible to better identify key kinases in beta-cells, develop new inhibitors, and evaluate the therapeutic repurposing of inhibitors already approved in clinical practice. The next years should allow us to know whether inhibitors of protein kinases will have a major impact in the treatment of diabetes.

## Figures and Tables

**Table 1:** Kinases as targets to protect beta-cell function and survival in diabetes.

Kinases	Inhibitors	Experimental Models and Development Stage	References
**Inflammation**			
MST1	Neratinib	In vitro, in vivo animal models, human islets	[29]
TAK1	5Z-7-oxozeaenol	In vitro, in vivo animal models	[30]
TPL2	1,7-naphthyridine-3-carbonitriles	In vitro, human islets	[31]
c-Abl	Imatinib/Gleevec®/Glivec	In vitro, in vivo animal models, clinical trial Phase II	[32,33]
TYK2	Structure-based drug design	In vitro, human islets	[34]
JAK1	ABT 317, LN3103801	In vitro, in vivo animal model	[35,36,55,56]
**Glucotoxicity and glulipotoxicity**			
GSK3β	Valproate, TDZD-8, KICG1338, Lithium	In vitro, in vivo animal models	[42,43,44,45]
IKKβ	BMS-345541	In vitro, in vivo animal models	[46]
MST1	Neratinib	In vitro, in vivo animal models, human islets	[29]
CK2 kinase	CX4945	In vitro, human islets	[47,48,49,50]
Glucokinase	d-mannoheptulose	In vitro	[51]
**Glucocorticoids**			
GSK3β	LiCl, SB216763	In vitro	[57]
